# ﻿DNA barcoding and morphology reveal European and western Asian *Arctiavillica* (Linnaeus, 1758) as a complex of species (Lepidoptera, Erebidae, Arctiinae)

**DOI:** 10.3897/zookeys.1159.95225

**Published:** 2023-04-25

**Authors:** Antonio S. Ortiz, Rosa M. Rubio, Josef J. de Freina, Juan J. Guerrero, Manuel Garre, José Luis Yela

**Affiliations:** 1 Department of Zoology and Physical Anthropology, University of Murcia, Campus de Espinardo, 30100 Murcia, Spain University of Murcia Murcia Spain; 2 Eduard Schmid-Str. 10, D-81541, München, Germany Unaffiliated Munich Germany; 3 Grupo DITEG, Área de Zoología, Facultad de Ciencias Ambientales, Universidad de Castilla-La Mancha, Avda, Carlos III, s.n., Campus Real Fábrica de Armas, E-45071, Toledo, Spain Universidad de Castilla-La Mancha Toledo Spain

**Keywords:** *Arctia*, COI, DNA barcoding, Europe, species delimitation, western Asia

## Abstract

Currently, the genus *Arctia* Schrank, 1802 includes approximately 16 species in the Palaearctic region, depending on the taxonomic interpretation. Here, populations of the *Arctiavillica* (Linnaeus, 1758) morphospecies complex were studied from Europe to the Middle East (Turkey, northern Iran) by molecular methods. Morphological treatment has traditionally revealed the presence of five nominal taxa: *A.villica* (Linnaeus, 1758), *A.angelica* (Boisduval, 1829), *A.konewkaii* (Freyer, 1831), *A.marchandi* de Freina, 1983, and *A.confluens* Romanoff, 1884. The molecular approach tests whether they represent well-delimited species. Subsequently, this study corroborates the suitability of the mitochondrial cytochrome c oxidase subunit 1 (COI) marker sequence for species delimitation. In total, 55 barcodes of the *Arctiavillica* complex were compared, and two molecular species delimitation algorithms were applied to reveal the potential Molecular Operational Taxonomic Units (MOTUs), namely the distance-based Barcode Index Number (BIN) System, and the hierarchical clustering algorithm based on a pairwise genetic distances approach using the Assemble Species by Automatic Partitioning (ASAP). The applied ASAP distance-based species delimitation method for the analysed dataset revealed an interspecific threshold of 2.0–3.5% K2P distance as suitable for species identification purposes of the Iberian *A.angelica* and the Sicilian *A.konewkaii* and less than 2% for the three taxa of the *A.villica* clade: *A.villica*, *A.confluens*, and *A.marchandi*. This study contributes to a better understanding of the taxonomy of the genus *Arctia* and challenges future revision of this genus in Turkey, the Caucasus, Transcaucasia as well as northern Iran using standard molecular markers.

## ﻿Introduction

The European fauna of Arctiinae moths (Erebidae) is particularly well-known comprising 113 species (Witt and Ronkay 2011), although some genera still require revision in terms of their composition and the taxonomic status of some species (e.g., *Setina* Schrank, 1802, *Eilema* Hübner, [1819], *Ocnogyna* Lederer, 1853, etc). Traditionally, the genus *Arctia* Schrank, 1802 was considered to include six or seven species in the West Palearctic region and ten species in the East Palearctic region divided into different species groups (*Arctia* Schrank, 1802, *Epicallia* Hübner, [1820], *Eucharia* Hübner [1820], and *Pericallia* Hübner, [1820]), depending on the taxonomic interpretation of the status for a number of taxa (de Freina and Witt 1987; Murzin 2003; Witt et al. 2011).

More recently, the use of eight molecular markers coupled with proper analytical algorithms (Maximum Likelihood and Bayesian Inference) has postulated a much more comprehensive view of the genus *Arctia* and closely related genera, including *Acerbia* Sotavalta, 1863, *Ammobiota* Wallengren, 1885, *Atlantarctia* Dubatolov, 1990, *Borearctia* Dubatolov, 1884, *Epicallia*, *Eucharia*, *Hyphoraia* Hübner, [1820], *Pararctia* Sotavalta, 1965, *Parasemia* Hübner, [1820], and *Pericallia* as synonyms or potential subgenera (Rönkä et al. 2016). Thus, the total number of species within the genus has significantly increased. Some of these *Arctia* species, such as *Arctiacaja* (Linnaeus, 1758), *A.festiva* (Hufnagel, 1766), *A.flavia* (Fuessly, 1779), and *A.villica* have a broad distribution from Europe to the Siberian mountains and steppes with several geographical subspecies and forms.

Following the current view, *Arctiavillica* is an extremely variable species in body size, coloration, wing pattern, and size of spots including the so far considered subspecies *A.v.villica* s.str., *A.v.angelica* (Boisduval, 1829), *A.v.konewkaii* (Freyer, 1831), *A.v.confluens* Romanoff, 1884 and *A.v.marchandi* de Freina, 1983 (Murzin 2003; Witt and Ronkay 2011). Murzin (2003) and Witt and Ronkay (2011) considered *A.villica* as having a West Palearctic distribution, occurring in the central and southern parts of Europe, from NW Africa and the Iberian Peninsula to Asia Minor, the Caucasus range and Transcaucasia, northern Iran and along the steppe belt to SW Siberia.

In the revisions of the *Arctiavillica* complex, de Freina and Nardelli (2007) and de Freina (2011) studied the nomenclature and systematics based on a large number of specimens from across their distribution range and concluded that all of its subspecies should be considered as species. *Arctiavillica* s. str. has a wide distribution from central and southern Europe to the Caucasus and west Siberia, *A.angelica* is restricted in the Iberian Peninsula and North Africa, *A.konewkaii* is endemic to Sicily, and *A.marchandi* and *A.confluens* are distributed from Transcaucasia and adjacent areas such as eastern Turkey, northern Syria, Iraq, and northern Iran to Central Asia. In the mentioned works, taxonomy and systematics based on external appearance and genital structures were reviewed, including eleven COI sequences from Caucasian and trans-Caucasian specimens of *A.villica*. Subsequently, Kizildağ et al. (2019) examined the taxonomic status of *A.marchandi* in southeastern Turkey. Their note, dealing with some barcodes of *A.villica* and *A.marchandi*, suggested subspecific status for *A.marchandi*, although the sequences were not published in GenBank or BOLD for their evaluation in later studies, which precludes any further comments.

The study of the morphological features and genitalia structures provides valuable criteria for species recognition according to de Freina and Nardelli (2007), de Freina (2011), and others. The recent integration of morphological and DNA-based approaches has proven to be a most effective way to advance species discovery and delineation (e.g., Lumley and Sperling 2010; Padial and De La Riva 2010; Schlick-Steiner et al. 2010), as well as to assist in detecting previously cryptic species (e.g., Hebert et al. 2004; Huemer and Hausmann 2009; Hausmann and Huemer 2011; Mutanen et al. 2012). Integration of molecular methods with morphological analyses may accelerate biodiversity inventories and corroborate the status of doubtful taxa (Smith et al. 2009).

In this paper, we present new insights from DNA barcodes of material collected along the geographical distribution of the different taxa of *Arctiavillica* complex to be added to the previous morphological studies in order to substantiate their taxonomic range.

## ﻿Materials and methods

### ﻿Abbreviations of specimen repositories

**MWM** Museum WITT, München (in ZSM);

**RCBA-UMU**Research Collection of Biología Animal, Universidad de Murcia;

**ZSM**Zoologische Staatssammlung München (SNSB).

### ﻿Morphological study

**Figure 1.**
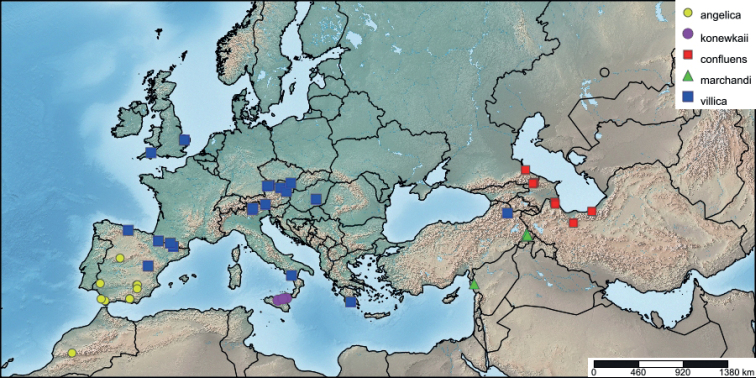
Distribution of *Arctia* samples sequenced. Note that each point may represent more than one specimen. The map was created using www.simplemappr.net.

This study is based on the results of the morphological study of a large amount of collected material deposited in the ZSM and MWM museums, developed by de Freina and Nardelli (2007) and de Freina (2011). New material was added and 115 adult specimens of the *Arctiavillica* complex (Fig. 1) were examined externally to evaluate possible differences in their colouration, spot size, and wing shape based on the taxonomic keys provided by de Freina and Nardelli (2007), Ylla et al. (2010), de Freina (2011) and Witt and Ronkay (2011), and were dissected using the standard procedure (Fibiger 1997) with minor modifications. Adult images were taken with a Nikon D70 digital camera and were z-stacked using Zerene software. Genital structures were examined using a Zeiss Stemi 508 stereomicroscope with a Zeiss Axiocam ICc5 digital camera and were compared with those published by de Freina and Nardelli (2007), de Freina (2011), and Witt and Ronkay (2011). Specimens were deposited in RCBA-UMU, in the Department of Zoology and Physical Anthropology of the University of Murcia (Spain), and in the Bavarian State Collection of Zoology in the ZSM (Germany).

### ﻿Molecular study

Thirty-four adult specimens of the *Arctiavillica* complex were processed and sequenced at the Canadian Centre for DNA Barcoding (CCDB, Guelph) to obtain DNA barcodes using the standard high-throughput protocol described by deWaard et al. (2008), which can be accessed at www.dnabarcoding.ca/pa/ge/research/protocols. The study was completed with publicly available sequences downloaded from The Barcode of Life (BOLD: www.boldsystem.org) (Ratnasingham and Hebert 2007). Ultimately, the analysis involved 55 *Arctiavillica* sensu lato COI sequences from Europe and countries around the Caucasus, with more than 500 bp for calculations and tree constructions (Table 1). Voucher data, GPS coordinates, images, sequences, GenBank accession numbers, and trace files are publicly available through the public data set (https://doi.org/10.5883/DS-VILLICA) in BOLD.

**Table 1.** Taxon names, old (BIN1) and present BINs (BIN2), BOLD accession numbers for the specimens used in distance estimations (Process ID), haplotype, and locality information (Country and Territory).

Taxon	BIN1	BIN2	Process ID	Haplotype	Country	Territory
*Arctiavillica*	ACP7520	AAC8627	ABOLA146-14	1	Austria	Kaernten
	ACP7520	AAC8627	ABOLC051-16	1	Austria	Niederöesterreich
	ACP7520	AAC8627	ABOLD441-16	1	Austria	Niederöesterreich
	ACP7520	AAC8627	FBLMZ558-12	1	Germany	Bavaria
	ACP7520	AAC8627	GWOSI563-10	1	Germany	Bavaria
	ACP7520	AAC8627	IBLAO1063-14	1	Hungary	Bacs-Kiskun
	ACP7520	AAC8627	NOENO403-17	1	Austria	Niederöesterreich
	ACP7520	AAC8627	LEASW136-19	2	Greece	Peloponnese
*Arctiamarchandi*	ACP7520	AAC8627	VNMB685-08	3	Syria	Aleppo
	AAC8627	AAC8627	IBLAO1080-14	4	Turkey	Hakkari
	AAC8627	AAC8627	VNMB690-08	4	Turkey	Hakkari
	AAC8627	AAC8627	VNMB689-08	5	Turkey	Hakkari
*Arctiavillica*	ACP7477	AAC8627	CGUKD205-09	6	U. Kingdom	Devon
	ACP7477	AAC8627	CGUKD277-09	6	U. Kingdom	Norfolk
*Arctiaangelica*	ACP7477	AAC8627	IBLAO1060-14	6	Spain	Castilla-León
	ACP7477	AAC8627	IBLAO1061-14	6	Spain	Castilla-León
*Arctiavillica*	ACP7477	AAC8627	IBLAO1073-14	6	Spain	Cataluña
	ACP7477	AAC8627	IBLAO1124-14	6	Spain	Aragon
	ACP7477	AAC8627	IBLAO1125-14	6	Spain	Asturias
	ACP7477	AAC8627	IBLAO1126-14	6	Spain	Asturias
	ACP7477	AAC8627	IBLAO951-14	6	Spain	Aragón
	ACP7477	AAC8627	IBLAO952-14	6	Spain	Aragón
	ACP7477	AAC8627	LEATC649-13	6	Italy	Südtirol
	ACP7477	AAC8627	GWORZ116-10	7	Italy	Basilicata
	ACP7477	AAC8627	IBLAO553-12	8	Spain	Cataluña
	ACP7477	AAC8627	PHLAC475-10	9	Italy	Südtirol
	ACP7477	AAC8627	VNMB688-08	10	Turkey	Kars
*Arctiaconfluens*	ACP7477	AAC8627	IBLAO1077-14	11	Azerbaijan	Masally
	ACP7477	AAC8627	IBLAO1078-14	11	Azerbaijan	Lerik
	ACP7477	AAC8627	VNMB686-08	11	Russia	Dagestan
	ACP7477	AAC8627	IBLAO1082-14	12	Russia	Dagestan
	ACP7477	AAC8627	IBLAO1081-14	13	Russia	Dagestan
	ACP7477	AAC8627	IBLAO1083-14	14	Russia	Dagestan
	ACP7477	AAC8627	VNMB687-08	15	Kyrgyzstan	Namagan
	ACP8428	AAC8627	IBLAO1064-14	16	Iran	Zanjan
*Arctiaangelica*	ABY6789	ABY6789	GBMIN80080-17	17	n/a	n/a
	ABY6789	ABY6789	IBLAO1068-14	17	Spain	Andalusia
	ABY6789	ABY6789	IBLAO1069-14	17	Spain	Andalusia
	ABY6789	ABY6789	IBLAO1070-14	17	Spain	Andalusia
	ABY6789	ABY6789	IBLAO1246-20	17	Spain	Andalusia
	ABY6789	ABY6789	IBLAO733-12	17	Spain	Andalusia
	ABY6789	ABY6789	IBLAO734-12	17	Spain	Andalusia
	ABY6789	ABY6789	IBLAO1067-14	18	Spain	Andalusia
	ABY6789	ABY6789	IBLAO1071-14	18	Spain	Castilla-La Mancha
	ABY6789	ABY6789	IBLAO1107-14	18	Spain	Castilla-La Mancha
	ABY6789	ABY6789	IBLAO1127-14	18	Spain	Castilla-La Mancha
	ABY6789	ABY6789	IBLAO1128-14	18	Spain	Castilla-La Mancha
	ABY6789	ABY6789	IBLAO1058-14	19	Morocco	Marrakech-Tensift-Al Hauz
	ABY6789	ABY6789	IBLAO1059-14	19	Morocco	Marrakech-Tensift-Al Hauz
*Arctiakonewkaii*	ACL5457	ACL5457	GBMIN80081-17	20	Italy	Sicily
	ACL5457	ACL5457	IBLAO1074-14	21	Italy	Sicily
*Arctiakonewkaii*	ACL5457	ACL5457	IBLAO1076-14	21	Italy	Sicily
	ACL5457	ACL5457	IBLAO1188-19	21	Italy	Sicily
	ACL5457	ACL5457	IBLAO1075-14	22	Italy	Sicily
*Arctiaconfluens*	AAC8628	AAC8628	VNMB693-08	23	Iran	Mazandaran
*Arctiacaja*		AAA8530	IBLAO551-12		Spain	Catalonia
*Arctiafestiva*		ABW9262	IBLAO1091-14		Spain	La Rioja
*Arctiaflavia*		AAV9830	ABOLA435-14		Austria	NordTirol
*Arctialapponica*		ACF2201	LON720-09		Norway	Sor-Varanger

Sequence divergences for the barcode region were calculated using the Kimura 2-parameter (K2P) model (Kimura 1980) and the degrees of interspecific genetic variation were calculated using the analytical tools of BOLD. All the new and public species sequences were downloaded and aligned with the CLUSTAL algorithm of the MEGA6 software (Tamura et al. 2013). Bootstrap values were calculated with 1000 replicates, and initial Neighbour-Joining (NJ) and Maximum Likelihood (ML) trees based on distance were constructed with the MEGA6 software. A phylogenetic hypothesis with Maximum Likelihood as an optimality criterion was generated using IQ-TREE v. 1.6.12 (Nguyen et al. 2015). An alignment of 658 bps for 59 samples was partitioned into codon positions with ModelFinder software (Kalyaanamoorthy et al. 2017) and the codon position was modelled with GTR+F. Support values were calculated by 1,000 replications of both ultrafast bootstraps (UFBoot; Hogan et al. 2017) and Shimodaira-Hasegawa-like approximate likelihood ratio test (SH-aLRT; Guindon et al. 2010), as well as approximate Bayes branch test (aBayes; Anisimova et al. 2011). We selected *Arctiaflavia*, *A.caja*, *A.festiva* and *A.lapponica* (Thunberg, 1791) which are taxonomically related to *A.villica* in the subtribe Arctiina, as outgroups to root the tree (Table 1). To assess the COI divergences between the taxa in the *A.villica* species complex and the other *Arctia* species from Europe, we included all sites with the pairwise deletion option. The number of haplotypes was calculated with DnaSP 5.10 software (Rozas et al. 2003).

To elucidate the taxonomic status of some of the *A.villica* species complex studied, two molecular species delimitation methods were applied to reveal the potential Molecular Operational Taxonomic Units (MOTUs) that could represent putative cryptic species. The two methods were distance-based: Barcode Index Number (BIN) System (Ratnasingham and Hebert 2013), and the hierarchical clustering algorithm that only considers a pairwise genetic distances approach using the Assemble Species by Automatic Partitioning (ASAP) (Puillandre et al. 2021).

The BIN method (Ratnasingham and Hebert 2013) is implemented as part of the Barcode of Life Data system (BOLD; Ratnasingham and Hebert 2007). Newly submitted sequences are compared together with sequences already available in BOLD. Sequences are clustered according to their molecular divergence using algorithms that aim at finding discontinuities between clusters and each cluster is assigned a globally unique and specific identifier Barcode Index Number (BIN) registered in BOLD.

The ASAP method (Puillandre et al. 2021) build species partitions from single locus sequence alignments (i.e., barcode data sets). Grounded in evolutionary theory, ASAP is the implementation of a hierarchical clustering algorithm that uses pairwise genetic distances, avoiding the computational burden of phylogenetic reconstruction and proposing species partitions by neglecting the use of biological a priori insight of intraspecific diversity. This method has greater potential than the other programs assessed, for instance the barcode-gap approach Automatic Barcode Gap Discovery (ABGD) (Puillandre et al. 2012), the General Mixed Yule-Coalescent model (GMYC) (Pons et al. 2006) and the Poisson Tree Process (PTP) (Zhang et al. 2013), both first developed in a Maximum Likelihood framework, and later extended to a Bayesian framework (Reid and Carstens 2012) according to Puillandre et al. (2021).

## ﻿Results

### ﻿Molecular analysis

In the dataset composed of 59 sequences, 55 specimens of the *A.villica* complex were sequenced or their conspecific sequences were acquired from the databases (BOLD) to analyse taxonomic identity and geographical species grouping, obtaining more than 572 bp for the barcode region (48 of them with 658 bp). In total, 23 different haplotypes were found in the 55 barcode sequences analysed of the five lineages of *A.villica* species complex ranging from eleven haplotypes in *A.villica* to three in *A.marchandi* (Table 1). The overall haplotype diversity (Hd) was 0.922±0.02 in 56 polymorphic sites and nucleotide diversity per site (Pi) was 0.01679. Nucleotide composition showed a A+T of 68.8%. No insertions or deletions were found.

**Figure 2.**
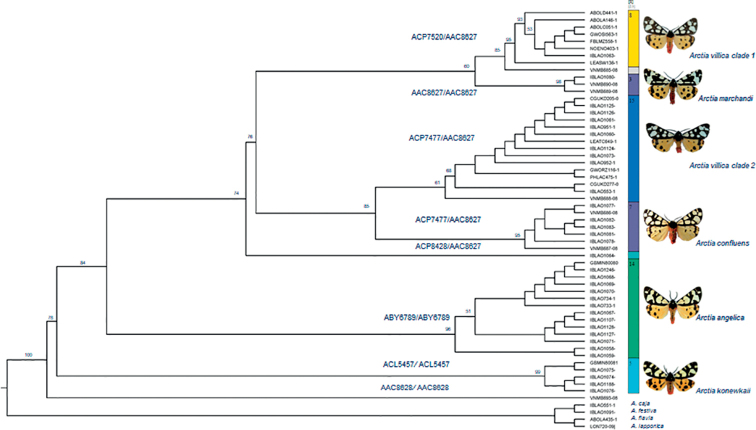
Maximum Likelihood tree of *Arctia* species, obtained from 59 nucleotide COI sequences. Bootstrap values > 50% are provided at major nodes. Old and new BINs are indicated for each node. ASAP species delimitation result is indicated by vertical and coloured bars.

Neighbour-Joining (NJ) and Maximum Likelihood (ML) trees of COI barcode region generated using MEGA software recovered the same topology and were practically identical, and all haplotypes could be unequivocally assigned to one of the eight major clades (Table 1). A phylogenetic hypothesis with ML as an optimality criterion was generated using IQ-TREE software and the topology obtained was chosen as the basis for our discussion with branch support values (Fig. 2). The monophyly of the *A.villica* species complex was recovered by all those methods and was corroborated with the inclusion of *A.flavia*, *A.caja*, *A.festiva*, and *A.lapponica* as outgroups to root the tree.

Three well-supported clades (bootstrap values higher than 70%) were found and were thereafter treated as three MOTUs considered as species, namely *A.villica*, *A.angelica*, and *A.konewkaii*. The divergence between these three groups varies between 2.4% and 3.5% (mean 2.8%; Table 2). The highest interspecific value was found between *A.villica* and *A.konewkaii* (3.5%) and between *A.angelica* and *A.konewkaii* (3.1%), whereas the lowest one was found between *A.villica* and *A.angelica* (2.4%). The total number of nucleotide substitutions between species is 56 variable sites (Table 2; a complete similarity matrix can be accessed in Suppl. material 1).

**Table 2.** Interspecific mean K2P (Kimura 2-Parameter) divergences (mean pairwise distances) based on the analysis of COI fragments (> 500 bp) among *Arctia* species.

	*A.angelica*	*A.confluens*	*A.konewkaii*	*A.marchandi*	*A.villica01*
*A.confluens*	2.7				
*A.konewkaii*	3.2	3.5			
*A.marchandi*	2.2	1.8	2.7		
*A.villica01*	2.4	1.9	3.3	1.3	
*A.villica02*	2.4	1.5	3.0	1.4	1.5

The *A.villica* clade shows five different sub-clades with genetic differences of less than 2% among them including the clade 1 sequences from Northern Spain, Italy, and the United Kingdom (15 samples with 6 haplotypes); the clade 2, with samples from Germany, Austria, and Hungary (7 samples with 1 haplotype); another clade with samples from Russia, Kyrgyzstan, and Azerbaijan (7 samples with 6 haplotypes); the Turkey clade (3 samples with 2 haplotypes); and the one from Syria (1 sample with one haplotype). West and Central European specimens initially identified as *A.villica* clades 1 and 2 differed up to 1.5% while *A.villica* clade 2 from Central Europe differed at 1.5% and 1.4% from *A.confluens* and *A.marchandi*, respectively (Table 2). Initially, the samples from Russia, Kyrgyzstan, and Azerbaijan were identified as *A.confluens*, and specimens from Turkey as *A.marchandi*, between which a difference of 1.8% was found. The highest interspecific values were found between both clades of *A.villica* and *A.marchandi* (1.8%) and *A.confluens* (1.5%), whereas the lowest one was found between both clades of *A.villica* and *A.marchandi* (1.4% and 1.3%) (Table 2). One specimen from Iran (IBLAO1064-14) identified as *A.confluens* differed up to 1.5% from the other subgroups.

### ﻿Delimitation of species

The mean K2P values between the morphologically determined species were used to study species delimitation using two different methods. The BIN System is an online framework that clusters barcode sequences algorithmically and is recalculated from time to time as the number of sequences of each species increases.

All the COI sequences from the five lineages were uploaded and examined into the Barcode of Life Data System (BOLD), resulting in four BINs (AAC8627, ABY6789, ACL5457, and AAC8628) (*n* = 55 seqs; 23 COI haplotypes). The BINAAC8627 was attributed to 35 sequences (15 COI haplotypes: 577–658 bp) from different continuous localities along Europe, from Spain and the United Kingdom, to Asia Minor, Caucasus, and Iran; ABY6789 was associated with 14 sequences, and three COI haplotypes (658 bp) located in the southern half of the Iberian Peninsula and Morocco; ACL5457 is unique from Sicily, with five sequences and three haplotypes (658 bp); and AAC8628 for one specimen identified as *A.confluens* (Table 1; field BIN2; Fig. 2). In previous recalculations, six different BINs were related to the five species with singles BIN for *A.angelica* (ABY6789), *A.konewkaii* (ACL5457), *A.marchandi* (AAC8627), two BINs for *A.villica* (ACP7520 and ACP7477) and three BINs for *A.confluens* (ACP7477, ACP8428, and AAC8628) sharing BINACP7477 with the *A.villica* clade 2 (Table 1; field BIN1; Fig. 2). This variation in the BIN values, suggesting the presence of different species with unique and specific identifier BIN within the studied *Arctiavillica* complex, should be updated with the results of this study.

ASAP method was performed on the data set of 54 sequences, representing all specimens sequenced. For the ASAP analysis, the sequence of a specimen identified as *A.confluens* from Iran (VNMB693-08; BIN: AAC8628) was excluded from further analysis because of the doubtful sequence that separates it from the rest of the groups. The analysis resulted in partitioning all COI sequences into eight MOTUs (hypothetical species) corresponding to five main lineages: *A.angelica*, *A.konewkaii*, *A.villica* (2 groups), *A.marchandi* (2 groups, one of them with only one barcode), and *A.confluens* (2 groups, one of them with only one barcode). ASAP score 2.5 was the smallest within the range of genetic distances and was calculated as the average among two values: the fourth largest p-value (0.255) and the smallest rank of relative barcode gap width (1.22e-04). The value of probability (p-value) quantifies the chances that each of its new groups represents a single species, and the rank calculates the width of the barcode gap between the previous and this new partition. Both metrics are combined into a single ASAP-score that is used to rank the partitions (see Puillandre et al. 2021). The graphical output shows each node of the hierarchical clustering with the same probability of being a panmictic species (p > 0.1) (Fig. 2).

The two methods used for species delimitation are congruent in recognizing the five main lineages as distinct from any other species studied. The exception are the specimens identified as *A.confluens*, since they are related to two different BINs: specimens collected from Iran with BINAAC8628 and the general *A.villica*BINAAC8627 including specimens from Caucasus and Syria. This high genetic variation needs to be further investigated with additional samples, particularly from Turkey, Caucasus, and Transcaucasia.

## ﻿Discussion

### ﻿Morphological and ecological traits

The contemporary species definitions and the properties upon which they are based were presented and discussed by e.g., de Queiroz (2007). Among them, biological, ecological, evolutionary, phylogenetic, phenotypic, and genotypic were noted as alternative species concepts, and a “Unified Species Concept” was presented by treating the species exists as a separately evolving metapopulation lineage and the definitions of species mentioned above as operational criteria for different lines of evidence to assessing lineage separation.

The *Arctiavillica* complex is a species group with high morphological and colour variability, characterized by the extensive black forewing background with small, well-defined, and rather distinct white patches partly or fully fused, but do not show clearly distinctive differences in the genitalia except in the slight variation of the aedeagus angle, although within each of the species, there is a wide range of variation of the characters with singular overlaps between the peripheric populations where introgression processes probably appear.

In this group of species, wing morphology, zoogeographical distribution, and maternal mitochondrial DNA (barcoding) are properties of lineage separation. Analysis of extensive samples of European and Asian populations of the *Arctiavillica* species complex based on morphological features and zoogeographical and biological information revealed the presence of five species, including different forms and varieties, named *A.villica*, *A.angelica*, *A.konewkaii*, *A.marchandi*, and *A.confluens* (de Freina and Nardelli 2007; de Freina 2011). Subsequently, Witt and Ronkay (2011) considered that their distinctive differences in morphology fit well into the range of variation of their characters and all the taxa were considered at subspecific taxonomic rank.

Nevertheless, de Freina and Nardelli (2007) noted that the genital morphology of the entire *A.villica* group is strikingly uniform in both sexes with an insignificant individual variability that is not relevant for the systematics and without signs of regionally deviating structures that could indicate existing subspecies or development processes leading to such. The absence of notable differences in the male and female genitalia, which is the basic feature of the biological species concept for interbreeding, reproductive isolation, and specific mate recognition or fertilization system does not constitute evidence contradicting a hypothesis of lineage separation since the lineage simply may not yet have evolved that properties, as might be expected if they are still in the divergence process, or do not need them because recognition among sexes is due to chemical mechanisms. There are many other such examples in the Noctuoidea (e.g., several species complexes in the genera *Euxoa* Hübner, [1821], *Agrotis* Ochsenheimer, 1816, *Cucullia* Schrank, 1802, *Apamea* Ochsenheimer, 1816, and so on).

Although the main criterion to separate Lepidoptera species is genital morphology, other pre- and post-reproductive isolation mechanisms must be considered that prevent the forthcoming of fertile offspring, even though mating may occur (e.g., Yela 2002). De Freina and Nardelli (2007) performed mating experiments between *A.villica* and *A.konewkaii*, from which a F1 was obtained without a fertile F2, suggesting that the reproductive barrier could be related to the pheromone chemistry and not to the genital structure. In the only case where mating was achieved between F2 specimens, the female spawned sterile eggs. The presence in nature of hybrid specimens with morphological characteristics of the male and barcoding of the female has been detected in the area where the populations of *A.villica* and *A.angelica* overlap in the Iberian Peninsula (Table 1; IBLAO1060-14 and IBLAO1061-14).

### ﻿BIN discrepancies and species

Detailed morphological, ecological, and genetic analysis can discriminate closely related species that show slight sequence divergence from their nearest neighbour. Molecular analyses enable initial biodiversity evaluation in such taxa, but there is no objective way to select the algorithm or input parameters that best recover actual species boundaries. In different groups of invertebrate taxa, a sequence divergence in the barcode region lower than 2% often corresponds to intraspecific differences, while higher values are typical of interspecific variation and recognized as distinct MOTUs (Hausmann et al. 2011). However, the divergence between young sister species may fall below the 2% threshold, while unusually variable species may exceed it. This is an immediate consequence of the gradual process of speciation, and nominal species do not always correspond to the same divergence stage.

The discrimination of divergences involving these young species requires more algorithmic finesse, and the selection of an effective algorithm for MOTU recognition is necessary. In our study, BIN and ASAP methods were selected to analyse the *Arctiavillica* complex sequences.

The Barcode Index Number (BIN) system is a persistent registry for animal MOTUs recognized through sequence variation in the barcode region. The BIN pipeline analyzes new sequence data for the barcode region as they are uploaded to BOLD, and BIN metadata are dynamic because key elements of specimen records on BOLD, especially taxonomic assignments, are frequently revised by data providers and because of the high flow of new records (Ratnasingham and Hebert 2013).

In 2017, BOLD calculated seven different BINS for the five species with singleton BINs for *A.angelica*, *A.konewkaii*, and *A.marchandi*, two for *A.villica* and three for *A.confluens*. Currently, four BINs have been calculated clearly differentiating *A.konewkaii* and *A.angelica*, while *A.villica* formed a group that includes *A.villica*, *A.marchandi*, and *A.confluens*. This variation in the BIN values suggests the presence of different species with unique and specific identifiers within the studied *Arctiavillica* complex.

This case of discordance between BIN assignments and the *Arctiavillica* species taxonomy proposed can reflect the inability of sequence variation at COI to diagnose species because of introgression or their young age. These “merged species” have diagnostic nucleotide substitutions in the barcode region that can be correlated with the morphological or ecological traits used in species diagnosis. BIN sharing can be made when algorithms used as ABGD (Automatic Barcode Gap Discovery, Puillandre et al. 2012), CROP (Clustering 16S rRNA for OTU Prediction; Hao et al. 2011), RESL (Refined Single Linkage; Ratnasingham and Hebert 2013), GMCY (Generalized Mixed Yule Coalescent; Fujisawa and Barraclough 2013), and jMOTU (Java Program to define MOTU; Jones et al. 2011) fail to partition very young species because of their limited sequence divergence that cannot be separated completely (Ratnasingham and Hebert 2013).

Dellicour and Flot (2018) noted that some methods generally perform well and mostly congruently providing similar species partitions inferred from independent data as other molecular markers, morphological data, or ecological data although they perform poorly when the number of sampled individuals per species or intra- versus interspecific divergences is too low (see Ahrens et al. 2016). Increased sample size and taxonomic and geographic coverage are critical to recognizing species boundaries from barcode sequence information, in addition to other species characteristics.

### ﻿Integration of molecular and morphological data

Concerning the species delimitation analyses, Puillandre et al. (2021) assessed ASAP power along with three other programs (ABGD, PTP, and GMYC) on real COI barcode data sets noticing the ASAP potential to become a relevant tool to test the hypothesis in an integrative taxonomy process. ASAP identifies a species partition ranked with different scores that must be subsequently tested against other evidence in an integrative taxonomy framework, especially when a single-locus data is used.

The ASAP procedure of the barcoding data obtained in our study indicates the existence of five *Arctia* species within the formerly known *Arctiavillica* subspecies distributed from the Iberia Peninsula to the areas around Transcaucasia and Iran. Two well-supported and reciprocally monophyletic groups were found in the Iberian Peninsula, *A.angelica*, and Sicily, *A.konewkaii*. The other group was made up by sequences of the polymorphic *A.villica* within two clusters which show notable sequence variability as expected from its wide geographic distribution across many European and Asian localities. These two groups represent two *A.villica* populations related to both species *A.marchandi* and *A.confluens*, respectively. The *A.villica* populations (clade 1) from Austria, Germany, and Hungary were related to the *A.marchandi* population from Turkey while one specimen from Syria identified as *A.villica* is equally related to both groups. The other *A.villica* population (clade 2) from Great Britain, Italy, and half of the Iberian Peninsula was related to the polymorphic *A.confluens* (Fig. 2). The polymorphic *A.confluens* group has three different BINs in the same area of distribution.

Hence, with the combined evidence from comparative morphological studies and the DNA barcode results presented above, *Arctia* species belonging to the *villica* group could be considered as metapopulations lineages separately evolving extended through time and including connected subpopulations according to the Unified Species Concept and Species Delimitation (de Queiroz, 2007). In our study, these *Arctia* species must have evolved separately from other lineages with separated historical biogeographic processes (Fig. 2).

The current distributions of *A.villica* species complex is likely to be the result of multiple range shifts driven by past climatic changes. Cyclic climatic change during the Pleistocene has caused repeated range shifts in most European taxa, profoundly influencing the biodiversity of Europe (for reviews, see Hewitt 1996, 1999, 2000, 2004; Taberlet et al. 1998; Schmitt 2007).

Three peninsular refugia in the south of the continent, Iberia, Italy, and Balkan harbour high biodiversity and endemism rates due in large part to their long-term environmental stability, which enables the persistence of palaeoendemic taxa (e.g., Jansson 2003; Graham et al. 2006). Isolation among refugial populations promotes genetic and phenotypic differentiation as a result of independent adaptation to local environments and genetic drift, with consequences for reproductive isolation between discrete refugial lineages and the creation of hybrid zones where diverged lineages come into secondary contact (Hewitt 1999).

The major lineage of *A.villica* may have diversified in four complexes represented by a widespread group of ancient *A.villica* from Spain to Russia and three species groups restricted to known areas in the Iberian Peninsula (*A.angelica*), Sicily (*A.konewkaii*), and around the Caucasus (*A.confluens* and *A.marchandi*), showing a pattern that could be considered evidence for similar ecological preferences or parallel histories for these species during the Quaternary. The fact that these four taxa with more restricted distribution make up separated clades to the broadly distributed *A.villica* suggests that all clades share it as their recent common ancestor. This phylogeographic pattern has been suggested for other Lepidoptera (e.g. those of the genera *Polyommatus* Latreille, 1804, *Erebia* Dalman, 1816, *Melitaea* Fabricius, 1807, *Parnassius* Latreille, 1804, *Chelis* Rambur, [1866]), in which it is postulated a marked role of climatic oscillations during the Pleistocene on population isolation and differentiation (Haubrich and Schmitt 2007; Albre et al. 2008; Lenevea et al. 2009; Vila et al. 2010; Todisco et al. 2012; Ortiz et al. 2015).

The apparent absence of a clear lineage sorting between *A.villica* and *A.confluens*-*A.marchandi* may be due to relatively recent radiation. Preliminary information in Kizildağ et al. (2019) suggested that *A.marchandi* should be considered at the subspecific level, although the *A.confluens*-*A.marchandi* divergence must be further evaluated when more specimens from all over their distributional ranges have been sequenced.

However, an increasing number of studies indicate that many endemic taxa inhabiting refugial regions are of Pleistocene origin and formed by allopatric fragmentation. In some cases, they are described as distinct species and, in other cases, these taxa are considered to be subspecies or lineages within species. The present occurrence of *A.angelica*, *A.confluens* and *A.marchandi* in the Iberian Peninsula and around the Caucasus, respectively, showing a sympatric distribution with *A.villica*, is thought to be the result of a range expansion of *A.villica* since the last glaciation events, due to the high ecological plasticity of this species and the finding of many suitable habitats, including *A.konewkaii* in Sicily.

Additional information from nuclear markers and a greater number of samples from the Caucasus will be crucial for the resolution of the different questions that are currently unresolved, such as *A.confluens* and *A.marchandi* intraspecific variability and whether they can be delimited and recognized as species utilizing integrated taxonomy.

## Supplementary materials

Supplementary material 1 Complete similarity matrix Data type Similarity index matrix Explanation note Complete similarity index matrix among *Arctiavillica* species. https://binary.pensoft.net/file/842094This dataset is made available under the Open Database License (http://opendatacommons.org/licenses/odbl/1.0/). The Open Database License (ODbL) is a license agreement intended to allow users to freely share, modify, and use this Dataset while maintaining this same freedom for others, provided that the original source and author(s) are credited.Antonio S. Ortiz, Rosa M. Rubio, Josef J. de Freina, Juan J. Guerrero, Manuel Garre, José Luis Yela
